# *Fez1/Lzts1 *a new mitotic regulator implicated in cancer development

**DOI:** 10.1186/1747-1028-2-24

**Published:** 2007-08-24

**Authors:** Andrea Vecchione, Carlo M Croce, Gustavo Baldassarre

**Affiliations:** 1 Department of Molecular Virology, Immunology and Medical Genetics and Comprehensive Cancer Center, Ohio State University, OH, USA; 2 Division of Anatomical Pathology, II Faculty of Medicine, University "La Sapienza", Ospedale Santo Andrea, Rome, Italy; 3 Division of Experimental Oncology 2 CRO-IRCCS, Aviano, Italy

## Abstract

Considerable evidence has accumulated suggesting that cancer has genetic origin, based on the development of genomic alterations, such as deletions, mutations, and/or methylations in critical genes for homeostasis of cellular functions, including cell survival, DNA replication and cell cycle control. Mechanism controlling the precise timing and sequence of cell cycle events as well as checkpoints insuring fidelity of those events are key targets that when disrupted could result in tumorigenesis. Mitosis is the process by which a cell duplicates its genetic information (DNA), in order to generate two, identical, daughter cells. In addition each daughter cell must receive one centrosome and the appropriate complements of cytoplasm and organelles. This process is conventionally divided in to five distinct stages: prophase, prometaphase, metaphase, anaphase and telophase that correspond to a different morphology of the cell. The entry into mitosis (M) is under the control of the cyclin dependent kinase Cdk1. During G2, the kinases Wee1 and Myt1 phosphorylate Cdk1 at T14/Y15 residues, rendering it inactive. The transition from G2 to M is promoted by the activation of Cdk1 via dephosphorylation by the Cdk1 phosphatase Cdc25C. Activated Cdk1 complexes translocate into the nucleus during prophase where phosphorylate numerous substrates in order to enhance their activation as the cells progresses trough prophase, prometaphase, and metaphase.

Recently we identified a new player: *FEZ1*/*LZTS1 *that contributes to the fine-tuning of the molecular events that determine progression through mitosis, and here will review its role in cancer development and in M phase regulation.

## Background

**Figure 1:**
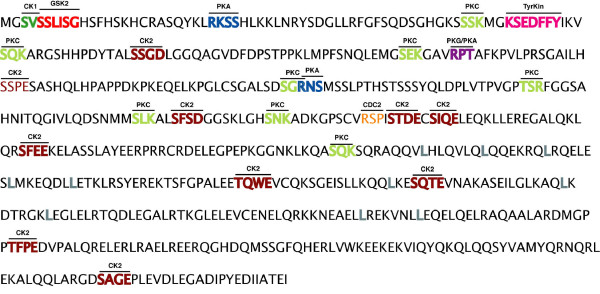
**Structural Functional Domains of Lzts1 protein**. Putative phosphorylation motifs of various kinases cAMP-dependent protein kinase A (PKA, in blue), protein kinase C (PKC, in green), cGMP-dependent kinase (PKG, in prugna), casein kinases I and II (CK1 and CK2, in green and in red, respectively), CDC2 kinase (CDC2, in orange), tyrosine kinase (TyrKin, in magenta). The Leucine residues of Leucine zipper motifs are indicated by the gray color.

Frequent loss of heterozygosity (LOH) at specific chromosomal regions in tumors implies the presence of tumor suppressor genes. One of these regions, located at the short arm of chromosome 8 (8p21.3–22) has been pointed out as frequently lost in different human malignancies as well as implicated in tumor progression. Investigation of this region, in primary esophageal cancer, has allowed the identification of the human *FEZ1/LZTS1 *(Leucine-Zipper Tumor Suppressor 1) gene at chromosome 8p22 [1]. *LZTS1 *gene show in non-cancerous cells a 6.8 kb mRNA including a 1.7 kb open reading frame (ORF), spanning exons 1–3, which encodes a 596-aa protein of 67 kDa [1]. Homology search of databases showed that the protein contains two Leucine-zipper motifs (Fig. 1) and it has 32% identity to the DNA binding domain of a cAmp-responsive activating transcription factor, Atf-5, although Lzts1 lacks the DNA recognition sub-domain usually found upstream of a Leucine-zipper motif in transcription factor. Moreover, searches for motifs and compositional analyses inside the Lzts1 protein sequence revealed the presence of multiple potential phosphorylation sites for different kinases (Fig. 1), such as PKA, CDC2 and PKC. The product of the *LZTS1 *gene is the founding member of a three member family proteins, all of which harbor a Leucine zipper Fez domain [2,3]. Interestingly protein alignment of the three members (Lzts1, Lzts2 and Lzts3) showed significant sequences similarity, particularly in the C-terminal potential Leucine zipper regions. Although recently has been reported [4] that the nuclear export sequence identified in Lzts2 is not conserved in both the Lzts1 and the Lzts3 proteins, suggesting a potential role for Lzts2 in the regulation of nuclear and cytoplasmic trafficking, which may reveal a different function for this family member.

### Expression pattern of LZTS1 in Primary Tumors and in Cancer Cell lines

Immunohistochemical studies of primary tumors indicated that Lzts1 protein expression in cancer cells is absent or markedly reduced in many different cancers compared with non-cancerous tissue [5-8]. Our previous studies showed that the expression of Lzts1 protein was undetectable or markedly reduced in 44% (39/88 cases) of primary gastric cancer with significant correlation to diffuse histotype (*P *< 0.001) [5], in 62% (37/60 cases) of primary transitional cell carcinoma of bladder with significant correlation to histopathological tumor grade (*P *< 0.005) [6], and in 64% (7/11 cases) of Bellini duct carcinoma [7]. Other studies showed that Lzts1 protein was lost or reduced in 68% (70/103 cases) of lung cancer with different histotypes, with a positive association with the tumor grade [8]. Overall, ~60% of different primary tumors showed loss or reduction of Lzts1 protein expression in cancer cells. Western blot and RT-PCR analyses in different cancer derived cell lines showed similar result [5-10], arguing that loss or reduction of LZTS1 is a frequent event in cancer development and progression. In addition LZTS1 is also mutated and/or deleted in several cancer types. We analyzed *LZTS1 *ORF (exon 1–3) in a total of 220 cancers, from 72 primary esophageal cancers, 18 esophageal cancer cell lines, 24 primary prostate cancers, 3 prostate cancer cell lines, 39 primary breast cancers, 25 breast cancer cell lines, 8 primary ovarian cancers, 4 leukemic cell lines, 1 cervical cancer cell line [1] and 26 primary gastric cancers [5], regardless of the presence or absence of *LZTS1 *expression. We found four point mutations in two primary esophageal cancers, in a prostate cancer cell line, and in a primary gastric carcinoma. In a primary esophageal tumor, alteration of TCC to CCC at codon 29 resulted in the substitution of Ser-29 with Pro-29, which is a predicted cAMP-dependent kinase phosphorylation site. In another primary esophageal cancer, alteration of AAG/Lys to GAG/Glu at codon 119 was found. Our LOH study indicated that these two patients had allelic losses at the *D8S261 *marker. Thus, these tumor cells retained the mutated *LZTS1 *allele and lost the normal *LZTS1 *allele by point mutation. The third point mutation was the change of CAG/Gln to TAG/Stop at codon 501 in a prostate cancer cell line, PC3, which resulted in coding of a putative 166-aa protein lacking the C terminus. The fourth mutation was a somatic missense mutation (CAC/His to CGC/Arg at *LZTS1 *codon 17) of one allele in a diffuse-type gastric carcinoma.

We identified, by RT-PCR, several internally truncated transcripts in esophageal cancer, prostate cancer, melanoma, and hematological malignancies (Table 1). The transcript from two independent esophageal cancers showed a frameshift within the ORF, which resulted in coding a short 76-aa protein [1]. We analyzed the nucleotide sequences around "breakpoints" of the deleted cDNAs and compared them to sequences from the full-length cDNA. The results showed that all of the acceptor sites contain the intronic AG flanking the exons, suggesting that the deleted transcripts in several tumors may be produced by alternative splicing.

**Table 1:** 

TUMOR	DELETION	RESULTS	AFFECTED EXONS	PUTATIVE PROTEIN CODED IN-FRAME
Esophagus	156–1542	FS	Exons 1,2,3	Leucine Zip(-)
Esophagus	558–1715	IF	Exons 2,3	Leucine Zip(-)
Esophagus	558–1715	IF	Exons 2,3	Leucine Zip(-)
Esophagus	558–1715	IF	Exons 2,3	Leucine Zip(-)
Esophagus	156–1542	FS	Exons 1,2,3	Leucine Zip(-)
	1402–1578	IF	Exon 3	Leucine Zip(+)
Prostate	1366–1641	IF	Exon 3	Leucine Zip(+)
	1402–1578	IF	Exon 3	Leucine Zip(+)
ALL	1402–1578	IF	Exon 3	Leucine Zip(+)
Melanoma	1417–1515	IF	Exon 3	Leucine Zip(+)
	1516–1584	IF	Exon 3	Leucine Zip(+)

**IF**, in-frame; **FS**, frameshift; **Leucine Zip(-)**, Lzts1 protein coded in-frame with Leucine zipper; **Leucine Zip(+)**, Lzts1 protein without Leucine zipper. ALL (acute lymphoblastic leukemia)

More recently a detailed DNA sequence analysis of *LZTS1 *was performed in a screening panel consisting of sporadic and hereditary prostate cancer (HPC) cases and unaffected controls. Twenty-four single nucleotide polymorphism (SNP), 15 of which were novel, were identified in germline DNA. Four coding SNP were identified. Eleven informative SNP were genotyped in 159 HPC probands, 245 sporadic prostate cancer cases, and 222 unaffected controls. Four of these SNP were statistically significant for association with prostate cancer (*P *< 0.04), supporting a role of *LZTS1 *in prostate cancer risk [11].

### Effect of *LZTS1 *Gene Introduction Into Cancer Cells

Experimental studies using different cancer derived cell lines from human bladder, breast and prostate tumor, showed that reintroduction of *LZTS1 *results in inhibition of tumor cell growth in vitro and/or tumorigenicity in vivo.

In one study, introduction of full-length *LZTS1 *cDNA resulted in suppression of tumorigenicity in nude mice, in the inhibition of stable colony-forming efficiencies of ~50% of highly metastatic rat prostate cells and human prostate and embryonic kidney cells, and in the reduction of cell growth with accumulation of cells at late S to G_2_/M stage of cell cycle [12]. In our studies, using breast cancer (MCF7) and urothelial cancer derived cell lines (SW780) we observed inhibition of cancer cell growth in vitro and reduced tumorigenicity in vivo. In these studies we pointed out that *LZTS1 *inhibits cancer cell growth through the regulation of mitosis, interacting with CDK1, an important factor for G_2_/M transition of cell cycle [13,6].

### *Lzts1 *is a tumor suppressor in mammals

To investigate the function of LZTS1 we generated *Lzts1 *Knockout mice (*Lzts1*^-/-^) [14]. In animals at the age of 8–24 months (average 16 months), pathological analysis demonstrated in animals either heterozygous or knockout the onset of spontaneous neoplasms. The neoplasms in *Lzts1*^-/- ^mice were breast tumors, hepatocellular carcinomas, lymphomas, soft tissue sarcomas, and lung adenomas, a spectrum suggesting that *Lzts1 *absence affects multiple cell types *in vivo*. In addition we used NMBA treatment to determine whether *Lzts1 *modulates carcinogen-induced malignancy development. Six weeks after NMBA administration all 20 *Lzts1*^-/- ^mice (100%) and all 33 *Lzts1*^+/- ^mice (100%) developed multiple tumors of the forestomach, whereas only 5 of 30 *Lzts1*^+/+ ^mice (17%) developed tumors (*Lzts1*^+/+ ^vs. *Lzts1*^-/-^, *P *< 0.001; *Lzts1*^+/+ ^vs. *Lzts1*^+/-^, *P *< 0.001).

Histological sections showed an array of lesions typically seen in NMBA-treated animals, including hyperplasia, focal hyperplastic lesions, papillomas and carcinomas. *Lzts1*^-/- ^and Lzts1^+/- ^mice developed at high frequency invasive carcinoma of the forestomach, while *Lzts1*^+/+ ^mostly showed thin and regular epithelia. All Lzts1^+/- ^mice retained the wild type allele that was negative for mutations, a finding suggesting haploinsufficiency of *Lzts1*.

### *Lzts1*: a novel regulator of the cell cycle

Biochemical investigation of the mouse *Lzts1 *gene using fibroblasts derived from *Lzts1*wt and null embryos (MEF), revealed that *Lzts1 *wt and null MEFs displayed a normal progression along the different phases of the cell cycle but showed a faster M phase progression associated to a lower Cyclin B1/Cdk1 activity [14]. Using video time-lapse microscopy, we were able to demonstrate that the faster M progression of *Lzts1*^-/- ^cells was due to the time they spent in prophase. The faster mitotic exit together with the lower Cdk1 activity resulted in an increase in the number of mitosis containing lagging chromosome with an increased hypertetraploid cell population [14]. The time to complete mitosis represents the key for proper DNA separation. Lzts1 absence decreases Cdk1 activity resulting in prompt mitotic exit accompanied by defects in DNA segregation. The mechanism whereby Lzts1 absence decreases Cdk1 activity was sought investigating the molecule responsible for Cdk1 activation: namely Cdc25C. Cdc25s family of phosphatase serves as a key activator of the Cdks/Cyclins [15]. The three mammals Cdc25 phosphatase (Cdc25A, B and C) are responsible for dephoshorylating Cdks/cyclins on pThr14 and/or pTyr15 residues. This dephosphorylation triggers the final activation of Cdks/cyclins activity during normal cell cycle progression.

Recently we discovered that mitotic *Lzts1*^-/- ^MEFs showed a higher Cdk1-pY15/Cdk1 ratio accompanied by lower levels of endogenous Cdc25C that results in a lower association between endogenous Cdc25C and endogenous Cdk1.

**Figure 2:**
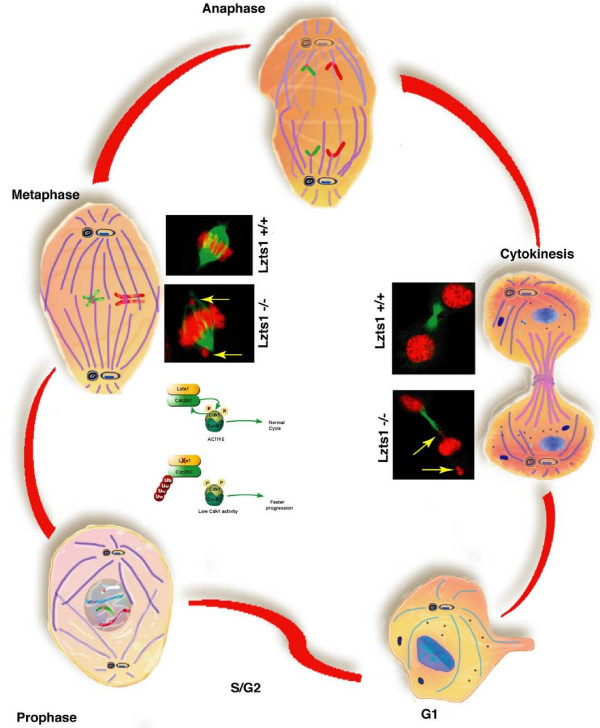
**Progression through the different stages of mitosis in *Lzts1*^+/+ ^and *Lzts1*^-/- ^cells**. During prophase the interaction between Lzts1 and Cdc25C allows high levels of Cdc25C expression and activity resulting in normal progression from prophase to metaphase. In Lzts1 deficient cell during prophase, Cdc25C is rapidly ubiquitinated and degraded thus determining a low activity of the cyclin B1/Cdk1 complex. As a consequence the cell progress faster through prophase and prometaphase and frequently undergo to chromosomes missegregation. Yellow arrows indicate lagging chromosomes in *Lzts1*^-/- ^cells.

It has been proposed that in G2/M arrested cells, Cdc25C may be efficiently ubiquitinated [16]. Therefore we tested whether Lzts1 might be involved in Cdc25C ubiquitination and we found that Lzts1 expression protects Cdc25C from proteosome degradation during M phase, thereby regulating Cdk1 activity [14] (Fig. 2).

This findings are rather surprising and change the classical view of M phase entrance and progression that implicates a central role for the high Cdk1 activity necessary for the proper execution of M phase [17-19].

It is possible that decreased Cdk1 activity by changes in its inhibitory phosphorylation during G2 phase may result in G2 block of the cell cycle, whereas decreasing Cdk1 activity in M phase before the establishment of the metaphase plate causes a faster prometaphase progression and mitotic exit, as in *Lzts1*^-/-^cells. In turn this event would lead to higher percentage of improper chromosome segregation resulting in cell transformation.

One intriguing observation is that Lzts1 absence seems to affects Cdc25C levels in mitotic MEFs but not in cells returned in G1 after the completion of cell division [14]. Accordingly, endogenous Lzts1 and Cdc25C proteins associated better during M phase than in proliferating cells extracts [14]. These observations suggest that in mitotic cells one or both proteins undergo to post-translational modifications or bind yet unidentified factors specifically expressed during M phase that could favor their interaction.

## Conclusion

Human tumor suppressors often function as negative regulator of the cell cycle. In fact their mutations can lead to failure of cell cycle checkpoint controls resulting in the accumulation of genetic changes contributing to a tumor phenotype. Several tumor suppressors are known to modulate the activities of Cdk/cyclin complexes. We focus here on a newly identified factor: Lzts1 which alterations lead to premature mitotic exit that results in chromosomal instability and aneuploidy. Being Lzts1 frequently down-regulated in many human tumors further investigations are needed in order to better understand its functions, eventually uncovering new opportunities for approaching cancer and other proliferation-related diseases.
